# Pancreatic β-cells respond to fuel pressure with an early metabolic switch

**DOI:** 10.1038/s41598-020-72348-1

**Published:** 2020-09-22

**Authors:** Ronja M. Malinowski, Seyed M. Ghiasi, Thomas Mandrup-Poulsen, Sebastian Meier, Mathilde H. Lerche, Jan H. Ardenkjær-Larsen, Pernille R. Jensen

**Affiliations:** 1 Department of Health Technology, Technical University of Denmark, Oersteds Pl. Bldg. 349, Room 120, 2800 Kgs. Lyngby, Denmark; 2 Department of Biomedical Sciences, University of Copenhagen, Copenhagen, Denmark; 3 Department of Metabolism, Digestion and Reproduction, Imperial College London, London, UK; 4 Department of Chemistry, Technical University of Denmark, Kgs. Lyngby, Denmark

**Keywords:** Metabolomics, Biochemistry, Biomarkers, Diabetes

## Abstract

Pancreatic β-cells become irreversibly damaged by long-term exposure to excessive glucose concentrations and lose their ability to carry out glucose stimulated insulin secretion (GSIS) upon damage. The β-cells are not able to control glucose uptake and they are therefore left vulnerable for endogenous toxicity from metabolites produced in excess amounts upon increased glucose availability. In order to handle excess fuel, the β-cells possess specific metabolic pathways, but little is known about these pathways. We present a study of β-cell metabolism under increased fuel pressure using a stable isotope resolved NMR approach to investigate early metabolic events leading up to β-cell dysfunction. The approach is based on a recently described combination of ^13^C metabolomics combined with signal enhanced NMR via dissolution dynamic nuclear polarization (dDNP). Glucose-responsive INS-1 β-cells were incubated with increasing concentrations of [U-^13^C] glucose under conditions where GSIS was not affected (2–8 h). We find that pyruvate and DHAP were the metabolites that responded most strongly to increasing fuel pressure. The two major divergence pathways for fuel excess, the glycerolipid/fatty acid metabolism and the polyol pathway, were found not only to operate at unchanged rate but also with similar quantity.

## Introduction

Glucotoxicity has been postulated as a pathogenic process contributing to pancreatic β-cell failure in type 2 diabetes^1,2^. Glucotoxicity can be defined as irreversible cell damage caused by chronic exposure to supraphysiological glucose concentrations^3^. The β-cells are designed to sense blood glucose in a narrow concentration range and respond with insulin secretion. Hence, the β-cell cannot shield itself from glucose toxicity by blocking glucose uptake, but relies on other metabolic protection mechanisms to avoid toxicity^4^. There are thus two different metabolic functions at play when the β-cells are exposed to excessive glucose concentration: (1) Metabolism of glucose and other nutrients that trigger insulin secretion. Multiple mechanisms are taking place via different metabolic products from glucose metabolism that generates the so-called metabolic coupling factors (MCF), a topic which remains incompletely understood^5^. (2) Initial handling of excessive fuel to avoid toxicity. It has been suggested that glucose carbons are diverted into glycerol release and lipid synthesis as a protective mechanism under excessive fuel exposure^6^. The polyol pathway has been reported to have a significant role for an oversupply of sorbitol in the eye lens of diabetic rats^7^, but recent reports also suggest an importance in pancreatic β -cells^8^. The interplay of these two aspects of glucose metabolism in β-cell function is yet to be fully understood and good biomarkers to investigate the interplay are missing.

Metabolomics offer a systems level view of cell metabolism and is well suited for unbiased investigations of cell metabolism. The method has been used in a number of studies investigating β-cell metabolism with mass spectrometry or nuclear magnetic resonance (NMR) as detection modalities^9^. Stable Isotope Resolved Metabolomics (SIRM) is an approach that measures isotope-filtered selection of molecules for elucidation of the dynamics and compartmentation of metabolic pathways and networks^10^. Fully ^13^C labelled glucose can for instance be used as a metabolic tracer to probe the NAD^+^-dependent oxidation of glucose to pyruvate in glycolysis. The metabolic transformations of the tracer lead to distinct labeling patterns in metabolic intermediates^11^. In this way, metabolic fluxes can be measured from temporal profiles of metabolite concentration changes. In conjunction with the use of stable isotope tracers, ^13^C-NMR is a method of choice for exploring metabolic reprogramming in major metabolic diseases such as cancer and diabetes^12,13^. ^13^C-NMR has a number of unparalleled advantages for metabolic studies, especially quantitativeness and reproducibility. In addition, NMR spectroscopy is a versatile method, whose information content can be tailored by transfer of magnetization between spins. The relative insensitivity of the method can be compensated by creating a non-equilibrium enhanced magnetization of the ^13^C nuclei by transfer of magnetization from electrons to the nuclei through dissolution dynamic nuclear polarization^14^. Such a combined method of hyperpolarized ^13^C-NMR for analysis of SIRM has recently been published^15^. In addition, the hyperpolarized NMR method is noninvasive and can be translated to preclinical and clinical in vivo studies, given that suitable biomarkers are found^16^.

In this study, we hypothesized that the hyperpolarized ^13^C NMR method can provide information on how the β-cells handle increased fuel pressure before reaching glucotoxicity. Our data show that weakly controlled carbon flux in glycolysis leads to an excess of ^13^C labelled pyruvate within the first two hours of incubation with [U-^13^C] glucose. A subsequent decrease in this metabolic end point of glycolysis indicates a switch in metabolism. Two known fuel surfeit pathways are shown not to respond to this unbalanced fuel pressure in β-cell metabolism.

## Results

### Hyperpolarized SIRM of β-cells

**Figure 1:**
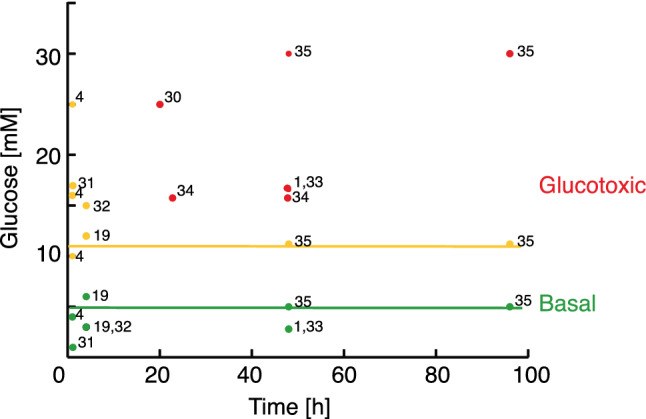
Literature survey of insulin response to glucose loading in β-cells. Green represents basal blood glucose concentration (up to 5 mM); yellow represent glucose concentrations, where insulin response is increased compared to the basal condition. The yellow line is drawn at 11.7 mM glucose; red represents glucotoxic conditions where insulin secretion is reduced compared to the basal condition. Numbers refer to literature references. Additional information including cell line and incubation conditions for the referred studies are collected in Tab S1.

In order to select conditions where β-cells are challenged by high fuel pressure before functional impairment, suitable exposure time and concentration of glucose need to be defined first. A literature survey was used to classify the functional state of β-cells based on their insulin release response to various levels of glucose load (time and concentration, Fig. 1). Basal glucose concentrations are within the range of 3–5 mM corresponding to normal blood glucose concentration. Up to a glucose concentration of 11.7 mM, insulin secretion was still increased compared to the basal level at long exposure time. Whereas the literature is sparse with respect to higher concentrations and short exposure times, it is clear that it is possible to drive the cells into a glucotoxic state with 17 mM glucose at long exposure times (24 h or longer), where insulin production is decreased compared to basal level. Accordingly, 11.7 mM and 17 mM glucose were chosen to represent non-glucotoxic and glucotoxic concentrations, respectively. Using a time frame of 2–8 h, the glucotoxic state is, however, not reached and metabolic effects can be considered early events before the onset of glucotoxicity (Fig. 1).

**Figure 2:**
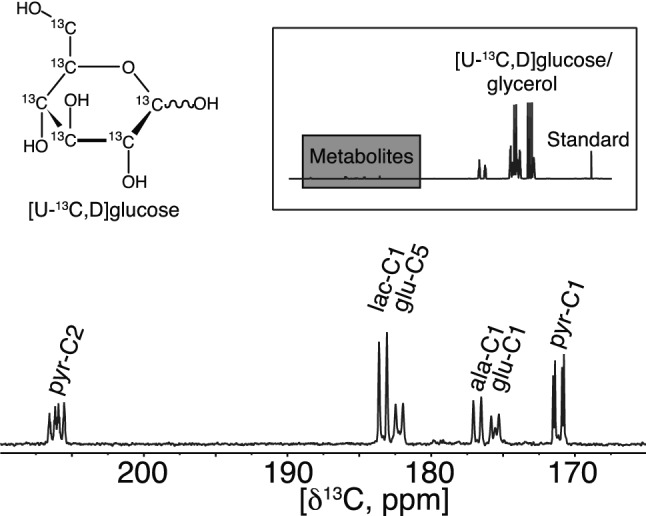
^13^C NMR spectrum of hyperpolarized metabolites after 4 h of incubation with 11.7 mM [U-^13^C_,_D] glucose. The metabolites were polarized for 90 min. after which they were subjected to rapid dissolution in hot phosphate buffer. Four metabolites were observed with distinct carbonyl shifts at: 2-^13^C-pyruvate (205.8 ppm), 1-^13^C-lactate (183.4 ppm), 5-^13^C-glutamate (182.2 ppm), 1-^13^C-alanine (176.8 ppm), 1-^13^C-glutamate (175.6 ppm) and 1-^13^C-pyruvate (171.2 ppm). The full spectrum is displayed in the insert where a grey box indicates the expanded region with the metabolites mentioned above. Other components in the sample were glucose, glycerol to mediate the hyperpolarization and an internal standard for quantification.

The metabolic fingerprint of INS-1 β-cells was obtained using hyperpolarized SIRM as recently described^15^ and an example of the carbonyl region from a 1D ^13^C spectrum from β-cells after 11.7 mM glucose incubation for 4 h is shown in Fig. 2. The inset shows the full spectrum with the signals from the uniformly labelled substrate. Glycerol was added to ease the hyperpolarization process and a signal of a reference was used for quantification. Glycerol and the reference were added ex *vivo* prior to analysis and do not affect the metabolic experiment. Observable carbonyls could be readily identified as 2-^13^C-pyruvate, (205.8 ppm), 1-^13^C-lactate (183.4 ppm), 5-^13^C-glutamate (182.2 ppm), 1-^13^C-alanine (176.8 ppm), 1-^13^C-glutamate (175.6 ppm) and 1-^13^C-pyruvate (171.2 ppm). The identification was based on the distinct chemical shifts of carbonyls (referenced to the internal standard) and the coupling constants originating from the ^13^C-^13^C couplings, since all metabolites are derived from uniformly ^13^C-labelled glucose.

### Effect of glucose concentration on metabolic fingerprint of β-cells

**Figure 3:**
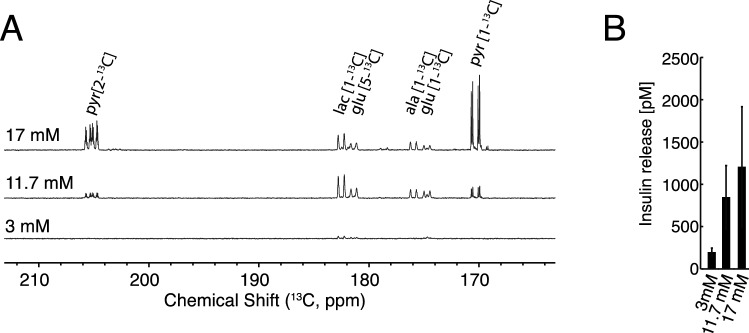
(**A**) ^13^C NMR spectrum of hyperpolarized metabolites after 4 h of incubation with 3, 11.7 and 17 mM [U-^13^C,D] glucose. The metabolites were polarized for 90 min after which they were subjected to rapid dissolution in hot phosphate buffer. (**B**) Corresponding accumulated insulin ELISA on extract supernatant and cell lysate from 3–17 mM glucose, n = 4. A statistically significant increase in insulin release was found by regression analysis (adjusted R = 0.999; ANOVA analysis p = 0.016). Insulin content was also determined (Fig S2).

To investigate the short term metabolic events under varying concentrations of glucose, INS-1 β-cells were incubated for 4 h with 3 mM, 11.7 mM and 17 mM levels of [U-^13^C,D] glucose. It is clear from a mere visual comparison of the 1D ^13^C NMR spectra that the metabolic fingerprint of β-cells responds to changes in glucose availability, and all measured metabolites increase with increased glucose availability (Fig. 3A).

**Figure 4:**
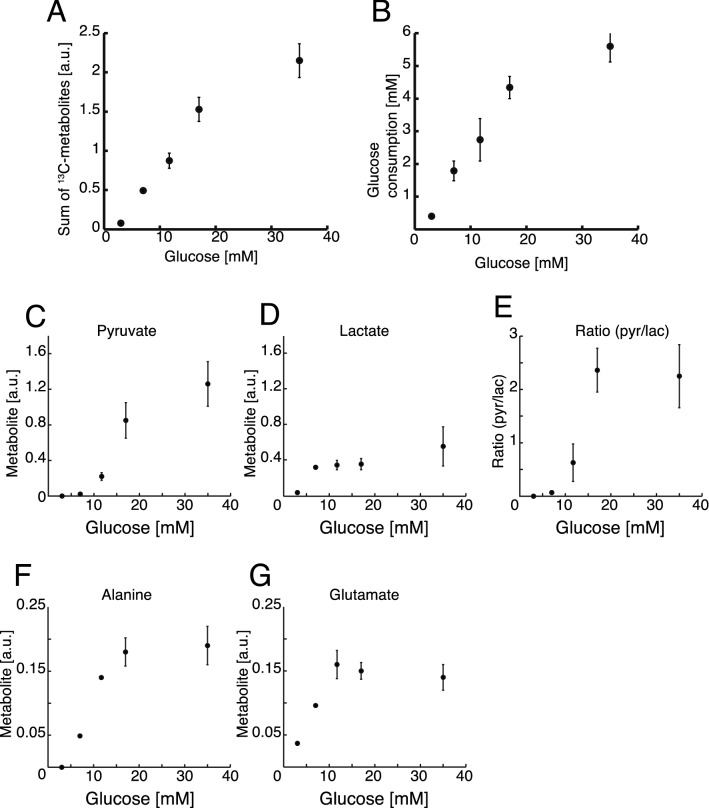
(**A**) Sum of measured metabolites relative to an internal standard plotted against the glucose concentration (3 – 35 mM) after 4 h of incubation, n = 4. (**B**) Glucose consumption (mM) plotted against the glucose concentration after 4 h of incubation measured by UV-assay, n = 4. (**C**–**G**) Individual metabolic profiles of ^13^C labelled metabolites contributing to the sum shown in A.

The accumulated insulin releases and insulin contents at the three glucose concentrations were measured from supernatants and cell pellets, respectively (Fig. 3B and Fig. S2 respectively). A statistically significant increase in insulin release was found by regression analysis (adjusted R = 0.999; ANOVA analysis p = 0.016). A dose–response curve showing the sum of all metabolites was measured for a larger range of concentrations between 3 and 35 mM glucose with an incubation time of 4 h (Fig. 4A). An increase in the sum of ^13^C-enriched metabolic products was observed up to 17 mM glucose. This increased metabolite production was directly reflected in an increased glucose consumption (Fig. 4B). At 35 mM glucose concentration, the β-cell metabolism stagnated, both with respect to metabolic products and with respect to glucose consumption. The individual metabolites followed the saturation behaviour but with different saturation points. Lactate saturated first at 7 mM glucose and pyruvate only at the highest concentration of 35 mM (Fig. 4C-G). The system is therefore not saturated for all metabolites unless very high glucose concentrations are used. This is in accordance with previously conducted studies^4^.

In summary, these data show that β-cells produce metabolites proportional to an increasing glucose uptake. Only at 35 mM glucose, the system is approaching saturation both in metabolite production and in glucose uptake.

### Effect of exposure time on metabolic fingerprint of β-cells

**Figure 5:**
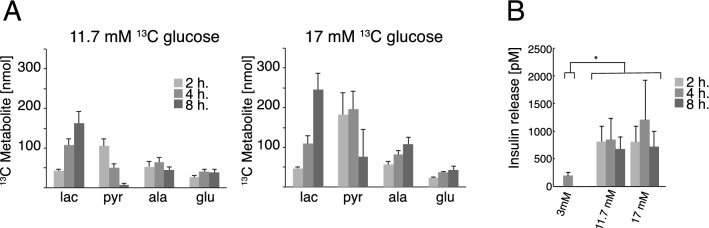
(**A**) Observed metabolites quantified relative to the internal standard. Cells were incubated for 2 – 8 h with respectively 11.7 mM and 17 mM [U-^13^C,D] glucose (n = 4). Lactate was significantly larger at 17 mM compared to 11.7 mM after 8 h (p < 0.05, Student’s t-test). Changes in glutamate and alanine with incubation time were not significant (p > 0.05) for 11.7 mM, whereas both glutamate (p = 0.01) and alanine (p = 0.002) levels increased significantly over time, if β-cells were exposed to 17 mM glucose (ANOVA). (**B**) Corresponding accumulated insulin release (n = 4). As expected, the insulin release was increased at the higher glucose levels (3 mM vs 11.7 mM and 17 mM; p < 0.001 (*). Insulin content was also determined (Fig S2).

Fuel pressure is the product of glucose concentration and exposure time. It was further investigated how the metabolic output changed with a 2–8 h incubation time, for the two glucose concentrations, 11.7 mM and 17 mM (Fig. 5A). Lactate was accumulating over time for both glucose concentrations. Accumulation of lactate was significantly larger at 17 mM compared to 11.7 mM after 8 h (p < 0.05). Changes in glutamate and alanine with incubation time were not significant (p > 0.05) for 11.7 mM, whereas both glutamate (p = 0.01) and alanine (p = 0.002) levels increased significantly over time, if β-cells were exposed to 17 mM glucose. An unexpected metabolic response was observed when the amount of pyruvate produced from the isotope labelled substrate was quantified over time. Initially, the pyruvate production increased to 106 ± 18 nmol (11.7 mM) and 182 ± 56 nmol (17 mM) at 2 h incubation time. Hereafter, however, this metabolite was depleted over time to reach 7 ± 4 nmol (11.7 mM) and 75 ± 70 nmol (17 mM) at 8 h incubation time. During the whole experiment period, the rate of glucose consumption was constant (0.52 mM/h for 11.7 mM and 0.68 mM/h for 17 mM) (Fig. S1). Accumulated insulin release and insulin content were measured for the same time points (Fig. 5B and Fig. S2 respectively). The insulin release was increased at the higher glucose levels (3 mM vs 11.7 mM and 17 mM; p < 0.001). There was no statistically significant change over time for 11.7 mM and 17 mM (ANOVA followed by Student’s t-tests).

**Figure 6:**
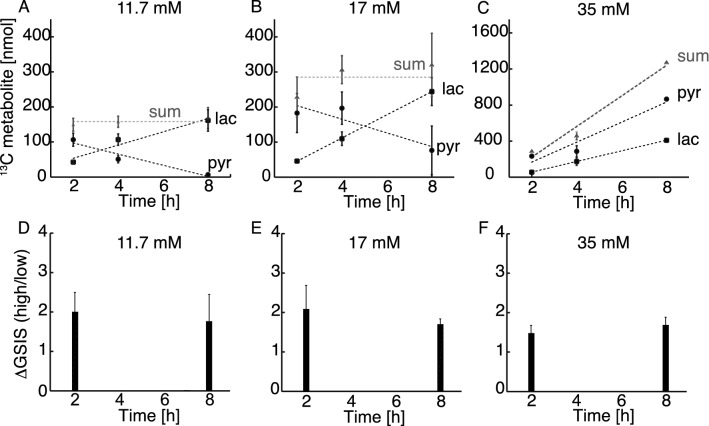
(**A–C**) The two main metabolites lactate and pyruvate and their sum responding over time to various glucose concentrations (11.7, 17 and 35 mM). The sum of pyruvate and lactate did not change significantly over time (p > 0.05, ANOVA followed by Student’s t-tests). (**D–F**) The corresponding glucose stimulated insulin secretion (GSIS) as a ratio between high and low glucose stimulation (2 and 17 mM glucose respectively) from GSIS experiment carried out after either 2 or 8 h of incubation with the three glucose concentrations (n = 4). No significant change was found, neither as function of time (2–8 h) nor concentration (11.7–35 mM) (p = 0.8 for 11.7 mM, p = 0.5 for 17 mM and p = 0.4 for 35 mM ΔGSIS 2 h vs 8 h, Student’s t-tests). The responses to incubations are similar for all conditions.

The sum of pyruvate and lactate accumulation over the time period 2–8 h did not change significantly with exposure to any of the glucose concentrations (158 ± 21 nmol for 11.7 mM and 285 ± 63 nmol for 17 mM, p > 0.05 for 2 vs 8 h incubation) indicating that a pseudo-steady state for the ^13^C-metabolites was established within 2 h and was sustained until 8 h (Fig. 6A and 6B). The individual amounts of pyruvate and lactate, on the other hand, did change over time. Lactate was produced with a constant rate of 0.31 nmol/min for 11.7 mM and 0.55 nmol/min for 17 mM. Pyruvate on the other hand was generated as the most abundant metabolite in the first 2 h, after which it decreased with the rate of 0.26 nmol/min for 11.7 mM and 0.33 nmol/min for 17 mM. The constant sum of ^13^C metabolites suggests that no metabolic bottlenecks appear in glycolysis upstream of pyruvate and that metabolic pathways at the intersection between glycolysis and energy metabolism do not change within the 8 h period studied. To investigate, whether it was possible to perturb this pseudo-steady state, the β-cells were challenged with a 35 mM glucose concentration for 2–8 h (Fig. 6C). At this glucose concentration, the sum of lactate and pyruvate metabolites was no longer constant over time, but increased significantly over time with a lactate production of 0.98 nmol/min and a pyruvate production of 1.85 nmol/min.

No significant change was found in glucose-stimulated insulin-secretion (GSIS), neither as function of time (2–8 h) nor concentration (11.7–35 mM) (Fig. 6D–F; p = 0.8 for 11.7 mM, p = 0.5 for 17 mM and p = 0.4 for 35 mM ΔGSIS 2 h vs 8 h). The responses to incubations are similar for all conditions. Despite of increased fuel pressure, the conditions were not glucotoxic on the 8 h time scale, since GSIS was not decreased. Also, no necrosis or advanced apoptosis was observed with Tryphan blue staining throughout the time course (Tab. S2).

Taken together, the data indicate that β-cells cannot avoid uptake of excess glucose load. However, they can accumulate pyruvate until a certain threshold, after which the cells start to switch metabolism to reduce the pyruvate pool. This ability is vanquished at very high glucose concentrations. All observed metabolic changes are taking place under non-glucotoxic conditions, as judged by the ability of the β-cells to secrete insulin.

### β-cells use an early metabolic switch under fuel pressure

**Figure 7:**
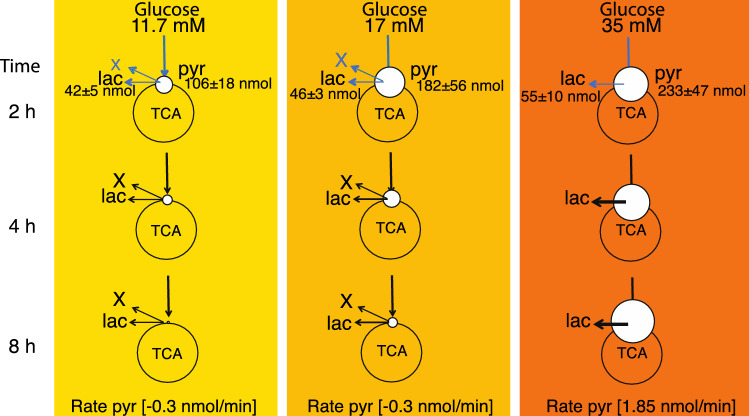
Schematic representation of β-cell metabolism under increasing fuel pressure (11.7 to 35 mM glucose) in the time span of 2–8 h. Measured amounts of pyruvate and lactate (nmol) after 2 h incubation are displayed for the three glucose concentrations. After 2 h incubation there is: Constant glucose consumption, constant production of glutamate (TCA cycle), constant production of lactate but large plasticity in the pyruvate pool (white circle). To account for the loss in observed pyruvate a divergence pathway away from the central glycolysis must take place. This divergence pathway (depicted as “X”) has the rate corresponding to the determined loss of pyruvate (0.3 nmol/min) for 11.7 and 17 mM glucose. At very high fuel pressure (35 mM) this metabolic pathway is not used anymore and pyruvate accumulates. Blue arrows indicate undetermined rates from 0–2 h. All data are scaled relative to the mildest conditions at 11.7 mM and 2 h for visualization (see Tab. S3 for actual numbers).

The observed metabolic rates of ^13^C glucose metabolism in β-cells for lactate and pyruvate did not change in the investigated concentration and time ranges (Fig. 6A–C). The corresponding glucose consumption was also constant (Fig. S1). After 2 h incubation, a transient accumulation of pyruvate occurred. Increased amounts of pyruvate were observed as a function of glucose concentration (increasing as 106, 182 and 233 nmol between 11.7, 17 and 35 mM glucose respectively), whereas the lactate production was relatively constant (42, 46 and 55 nmol for 11.7, 17 and 35 mM glucose respectively) (Fig. 7). At prolonged exposure times, the rate of pyruvate production increased as a function of glucose concentration from −0.3 nmol/min (for 11.7 and 17 mM glucose) to 1.85 nmol/min at 35 mM glucose concentration (Figs. 6, 7). Because lactate production, TCA cycle and glucose consumption between 11.7 and 17 mM glucose concentration were stable, there have to be other active metabolic pathways, which explains the observed depletion of pyruvate. This rationale indicates a switch in β-cell metabolism between an early stage, where fuel pressure results in pyruvate accumulation (0–2 h), and a later stage where the pyruvate pool is decreased. Notably, the metabolic events occur even though conditions were not glucotoxic to the β-cells, since GSIS was not decreased (Fig. 6D–F) and no necrosis or advanced apoptosis as observed with Tryphan blue staining could be observed (Tab. S2). Hence, spectroscopic methods potentially provide metabolic biomarkers prior to alterations in morphology, glucose consumption and insulin secretion.

### Fuel surfeit pathways in β-cells

**Figure 8:**
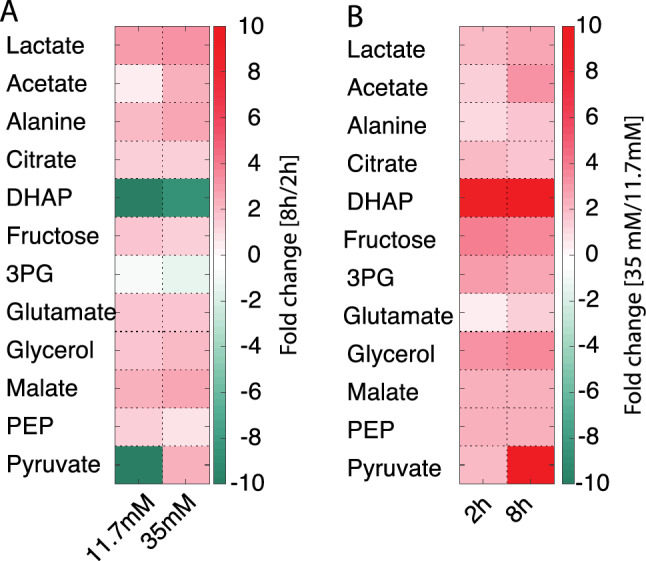
Colour map of metabolites originating from [U-^13^C] glucose measured in ^1^H-^13^C-HSQC NMR spectra as mean of n = 3 replica. (**A**) For each metabolite, the ratio of concentrations between determinations after 8 h and 2 h incubation is shown. (**B**) For each metabolite, the ratio between 35 mM and 11.7 mM glucose is shown. Individual ratios with standard deviation are given in Fig S3.

A more classic SIRM NMR approach was taken to obtain information from additional metabolites in the pathways around the central carbon metabolism. Two-dimensional ^1^H-^13^C HSQC NMR spectra were recorded for the highest and lowest time points and concentrations (11.7 mM glucose at 2 h and 8 h together with 35 mM glucose at 2 h and 8 h). Especially, fuel surfeit products like glycerol and fructose were of interest. In addition to the four metabolites obtained with hyperpolarized NMR, eight metabolic intermediates were quantified (acetate, citrate, dihydroxyacetonephosphate (DHAP), fructose, 3-phosphoglycerate (3PG), glycerol, malate and phosphoenolpyruvate (PEP)). With the exception of pyruvate, 3PG and DHAP, all metabolites increased to some extent, but less than threefold including the TCA cycle intermediates citrate and malate (Fig. 8A). 3PG displayed a ratio very close to unity whereas pyruvate and DHAP decreased with a factor of more than 10 (Fig. 8). Two end products of fuel surfeit pathways were observed, namely fructose and glycerol. Fructose is produced via the polyol pathway, which is a comparatively overlooked pathway involved in handling glucose overflow^8,17^. In this pathway, glucose is reduced to sorbitol and subsequently reoxidized to fructose using NADPH and NAD^+^ as co-substrates in the two steps, which result in a net aldose to ketose isomerization. The formation of fructose was increased similarly over time at different glucose concentrations (1.7 ± 0.6 and 1.5 ± 0.3-fold at 11.7 and 35 mM glucose, respectively). Likewise, glycerol formation increased similarly over time for both glucose concentrations (1.7 ± 0.1 and 1.9 ± 0.6-fold at 11.7 and 35 mM glucose, respectively). The two end products of known fuel surfeit pathways thus do not increase significantly with increasing glucose concentration. ^1^H-^13^C HSQC data are rarely used for absolute quantification due to sensitivity to parameters such as transfer delays and relaxation times. These variations are collected in a response factor that has to be determined for each individual metabolite^18^. In addition, the number of protons bound to the carbon must be accounted for if signals from different metabolites are compared. An estimate of the production of the metabolites based solely on proton content revealed that metabolites produced after 8 h at a glucose concentration of 11.7 mM were dominated by lactate, alanine and glutamate, consistent with the observations using dDNP-NMR data. Other metabolites formed from the substrate at significant amount included glycerol and fructose, which were produced in similar amounts (Fig. S4), thus indicating a similar flux through these two fuel surfeit pathways. This observation was confirmed by comparison to reference HSQC spectra with different known amounts of the two metabolites. By this method the ratio between produced glycerol and fructose was determined to be 1.3 ± 0.2.

Time dependent changes of metabolite concentrations are means to identifying metabolites involved in metabolic bottlenecks. Classically, dose dependence is investigated at fixed time points (2 h and 8 h in this study)^1,4,5,19^. This approach is shown in Fig. 8B. Cell homeostasis was maintained for all metabolites except for DHAP, and changes below a factor of three were observed after 2 h. Only after 8 h, pyruvate, PEP, 3PG and DHAP responded with an increase above three-fold. Overall, pyruvate and DHAP were the two metabolites that responded strongest to the extra cellular glucose concentration employed herein. The metabolic conversion of pyruvate and DHAP both use NADH for subsequent reduction to lactate and glycerolphosphate, respectively (Fig. S5). These reactions have previously been shown to respond to redox changes in yeast and mammalian cancer cells^20,21^. Since these metabolic pathways are part of the strongly conserved central carbon metabolism we suggest that a similar mechanism might be used as defense mechanism in β-cells, as well.

Overall, pyruvate and DHAP were identified as the two metabolites with the largest alteration as response to glucose metabolism. Most other metabolites were accumulated moderately in response to glucose. This includes end products of two fuel surfeit pathways, glycerol and fructose. Absolute quantification of glycerol and fructose revealed that they were formed at comparable amounts.

## Discussion

We have studied the energy metabolism of β-cells exposed to glucose concentrations ranging from 3–35 mM over the time course of 2–8 h with the aim of investigating early metabolic events of excess fuel handling. Our study was performed with SIRM in combination with hyperpolarized ^13^C NMR. The method showed high sensitivity and specificity in addition to permitting absolute quantifications of energy metabolites. An increased sensitivity obtained by hyperpolarized ^13^C-NMR made direct detection of ^13^C-isotope enriched metabolites possible for time- resolved β-cell metabolism. The employed ^13^C-isotopically enriched glucose allowed an unambiguous identification of the glycolytic end-product, pyruvate. Evidence for lactate dehydrogenase and transaminase activity was obtained through the identification of isotope enriched lactate and alanine. Mitochondrial activity was confirmed by the presence of isotope enriched glutamate. An approach using ^1^H-^13^C HSQC spectra to track relative isotope enriched metabolite levels was used to gain insight to regulation of fuel surfeit pathways together with important TCA cycle intermediates.

We observed a significant accumulation of pyruvate over glucose concentrations ranging from 3 to 35 mM. The pyruvate pool increased 5.7-fold between 11.7 mM and 35 mM external glucose concentration and 4 h exposure time. The increase was even more pronounced at longer exposure times of 8 h and reached more than 100-fold, as pyruvate concentrations decrease over time at 11.7 mM glucose concentration, but increases over time at 35 mM external glucose concentration. An increase in the pyruvate pool was previously observed, if β-cells were exposed to increasing glucose concentration between 2.8 mM and 16.7 mM at short exposure times (1 h)^4^ and at long exposure times (48 h)^1^. Label influx into the pyruvate pool starts at time point zero and reaches a maximum within approximately 2–4 h at 11.7 mM and 17 mM glucose concentration (Fig. 6 A,B), whereupon the labelled pyruvate pool decreases. This switch in ^13^C flux as revealed by the changed pyruvate metabolism over time has to our knowledge not been reported before.

Pyruvate is not considered a direct metabolic coupling factor (MCF) for insulin response, since pyruvate cannot increase GSIS^22^. Pyruvate cycling indirectly generates NADPH, which is considered a MCF, since it correlates with GSIS^19,23^. In a study searching for metabolic pathways that are important for excess fuel handling, Mugabo et al. screened for metabolites that did not correlate with insulin secretion but continued to increase above concentrations of 17 mM glucose^4^. They found that most metabolites correlated well with GSIS with a slight increase between 11 and 20 mM. Glycerol metabolism in particular was described as a fuel surfeit pathway that fulfilled the criterion of enhanced flux beyond 17 mM glucose concentration. In our study, metabolic patterns correlate with the observations of Mugabo et al., when interpreting the data in a dose dependent manner. This cross-laboratory reproducibility is encouraging, since different extraction protocols and analytical methods had been reported to skew data interpretation in other studies^24^.

Here we observe a second important fuel surfeit pathway, which is used to a similar extent as the glycerol pathway, namely the polyol pathway with fructose as end product. Only recently has this pathway been discussed as significant in pancreatic β -cells^8,17,25^. The metabolites in the two fuel surfeit pathways respond to glucose with an increase, similar to most other observed metabolites, and are as such not releasing the excess-fuel detoxification in β-cells. In this context, it should be pointed out that the cellular model system (INS-1) used in this study contains higher LDH activity than primary β-cells. The higher lactate production in this system release the pressure on the TCA cycle in a similar way as the fuel-surfeit pathways discussed here and thus might even diminish the need for fuel-surfeit pathways. The fuel-surfeit pathways are observed to operate at unchanged capacity from 11.7 to 35 mM glucose. We thus do not predict the contribution of LDH activity to interfere, since LDH activity is saturated at 7 mM glucose.

In normally functioning β-cells, NADH formed from glycolysis will not be lost in the reduction of pyruvate to lactate, but will rather be transferred to the mitochondria as a substrate of the respiratory chain^26^. This transfer assures a tight coupling between glycolysis and mitochondrial metabolism essential for glucose sensing. Under glucose load up to 17 mM (Fig. 7), our data show a constant glucose consumption and initial accumulation of pyruvate, which is a net oxidation and leads to an accumulation of NADH. This excess of NADH is not reduced by lactate dehydrogenase (LDH), as witnessed by a constant rate of lactate production. Likewise, the TCA cycle activity is less affected over time as observed by low increase in glutamate, citrate and malate formation. Therefore, another NADH dependent reaction must take place in the β-cell. This diverging pathway is reflected by the herein observed decreased pyruvate production rate.

Despite of great efforts to understand the β-cell metabolism, it remains unclear, how the overproduction of NADH is handled. Various mechanisms for maintaining redox balance in β -cells have been discussed. The active NADH shuttle system via the glycolytic enzyme glyceraldehyde 3-phosphate (GAPDH) participates in the coupling between the cytosol and mitochondria by transferring NADH^27^. The cytosolic glycerolipid/fatty acid cycle operates via DHAP and NADH. In addition, the mitochondrial Complex 1 has been suggested as a key-player for maintaining redox balance in β-cells^25^. Definitive answers as to how NADH is handled in β-cells are beyond the scope of the current study. On the hour time scale, a divergence pathway is upregulated in normally functioning β-cell, as revealed by the decrease in isotope enriched pyruvate.

The chosen glucose concentration in this study never resulted in glucotoxicity, since no decreased insulin response was observed, likely due to the short exposure time. The studied metabolic changes were thus prior to the failure of the β-cell. Most reported studies focus on one time point and several glucose concentrations. However, when we studied metabolism over time, it became evident that an initially accumulated pyruvate pool will diminish, as a diverging metabolic pathway is up regulated on the hour time scale. Importantly, this initial regulation thus takes place before mitochondrial processes affect insulin secretion.

In conclusion, this study identifies pyruvate as the most responsive major metabolite in β-cell metabolism under fuel pressure. Pyruvate is generated in amounts large enough for detection with dDNP-NMR. As a central metabolite in the junction between glycolysis and the TCA cycle it’s metabolic fate impacts many aspects of cellular biochemistry. An initial metabolic switch is observed before a change in insulin secretion is manifested. The end products of the two best-described fuel-surfeit pathways, glycerol and fructose do not respond to the metabolic switch revealed by pyruvate. Further studies are needed to elucidate the mechanisms responsible for this switch. Hyperpolarized NMR is a noninvasive method that can be translated to preclinical and clinical in vivo studies. The current study gives reason to hope that metabolic changes may become detectable in vivo prior to glucotoxicity through hyperpolarized NMR.

## Experimental procedures

### Cell culture

INS-1 cells [obtained from Prof. Claes B. Wollheim] were cultured in Gibco RPMI 1,640 Medium, GlutaMAX Suplement (Thermo Fisher Scientific) with 10% Fetal Bovine Serum (FBS) (Sigma-Aldrich), 1% Penicillin–Streptomycin (Sigma-Aldrich) and 500 μL of 50 mM 2-Mercaptoethanol (Thermo Fisher Scientific). This culture medium contains 11 mM glucose. The culture was maintained at 37 ºC under humidified (5% CO_2_, 95% air) conditions. Cell number and viability were measured by an automatic cell counter (EVE, NanoEntek). Cells were subcultured in 175 cm^2^ flasks at a density of 3·10^6^ cells and grown for 7 days with fresh medium supplied every 2–3 days.

### Glucose-stimulated insulin secretion (GSIS)

Cells were seeded in a 24-well plate (0.3·10^6^) 48 h prior to the experiment. Initially, the cells were pre-incubated at various concentrations (7–35 mM glucose in 40 mM phosphate buffer) for 2–8 h. The media were discarded, and the cells were incubated with 2 mM glucose in Krebs Ringer Hepes Buffer (KRHB) (NaCl (120 mM), KCl (5 mM), CaCl (2 mM), MgCl (1 mM), NaHCO_3_ (25 mM), HEPES (5.5 mM)) for 1 h. After this, GSIS was performed (1 h at 2 mM glucose, then 1 h 16.7 mM glucose in KRHB), all supernatants were collected for ELISA, and the cells were finally lysed with NP-40 buffer (Life Technologies) for insulin content measurements.

### Insulin ELISA

Supernatants (1:1000 dilution for accumulated insulin release and 1:100 for GSIS experiments) and cell lysate (1:2000 dilution) were used for measurement in duplicates of accumulated insulin and insulin content, respectively, using competitive insulin ELISA assay^28^ with the modification that an enzyme substrate 1-step Ultra TMB (3,3′,5,5′-tetramethylbenzidine) (Life Technologies) was used here.

### Metabolism experiments

Cells were trypsinized and counted. Ten million cells were placed in 167 μL phosphate-buffer (40 mM, pH 7.4) in an Eppendorf tube. Phosphate-buffer containing the isotope labelled substrate [U-^13^C,D] glucose or [U-^13^C] glucose (Sigma Aldrich) was added (333 μL) and the Eppendorf tube was placed on a thermomixer (Hettich Benelux) at 37 °C, 500 rpm, for the period of time indicated. The reaction was stopped by addition of 200 μL of ice-cold 2.2 M perchloric acid (for hyperpolarization experiments) or by immersing the tube in liquid nitrogen (for glucose utilization). The extracts for hyperpolarization were processed as described in^15^ and freeze-dried.

### Glucose utilization

The glucose consumption was measured on a parallel set of samples from the above metabolism experiment. At the indicated time points the samples were snap frozen in liquid nitrogen to stop metabolism. All samples were then heated to 65 °C for 5 min followed by centrifugation at 10,000 rpm, for 10 min. The supernatants were collected in clean Eppendorf tubes and the concentration of glucose was hereafter determined using Glucose (GO) Assay Kit (Sigma Aldrich), with a standard curve based on [U-^13^C,D] glucose. Absorbance was measured at wavelength 540 nm on a BioTek EPOCH 2 microplate reader (Holm & Halby, Brøndby, Denmark).

### Hyperpolarization and NMR acquisition

The freeze-dried extract was dissolved in 50 μL ultra-pure water, mixed with 92.5 mg polarization medium (967 mg glycerol, 38 mg OX063, 2.7 mM Gd-complex and 5 μL 50 mM [1-^13^C] HP001 (1.1-bis (hydroxymethyl) cyclopropane) was used as internal reference. The sample was polarized for 1.5 h on HyperSense (Oxford Instruments), after which it was dissolved in 5 mL phosphate buffer (40 mM, EDTA 100 mg/L) brought to a temperature of 175 ºC. The dissolved sample was quickly transferred to a 400 MHz NMR (Agilent Technologies, USA). Acquisition started 10 s post dissolution using a 90º flip angle and no ^1^H decoupling. The data were analysed using TopSpin 3.2 (Bruker).

### NMR metabolomics

The freeze-dried extract was dissolved in 550 μL phosphate buffer (20 mM, pH 7.5 in D_2_O). ^1^H-^13^C HSQC spectra were obtained on an 800 MHz Avance III NMR spectrometer (Bruker, Switzerland) with a TCI z-gradient CryoProbe. 2048 and 512 complex data points were acquired in the ^1^H and ^13^C dimensions to sample the FID for 160 and 21 ms, respectively. Due to the transfer of magnetization from ^1^H to ^13^C in the ^1^H-^13^C-HSQC experiment, ^13^C labelled metabolites are preferentially detected in the experiment. Thus, an increased selectivity for the detection of metabolites from the ^13^C labelled substrate glucose compared to background metabolites (with natural abundance of ~ 1% ^13^C) can be achieved.

The response factors of glycerol and fructose were determined from three individually made samples consisting of the following amounts: Glycerol 9.1; 5.0; 4.9 mg, fructose 9.6; 9.6; 18.2 mg. Each sample was dissolved in 5 ml of the same buffer as used for the metabolite samples. ^1^H-^13^C HSQC spectra were acquired in the same way as for the biological samples. Integrals for the same peaks as used for the metabolite samples were evaluated and the signal ratio between glycerol and fructose was determined to 2.5 ± 0.06. This difference in peak response factor was used to quantify glycerol and fructose in the biological samples.

The metabolites were assigned using a combination of a database approach based on Metabolab^29^ and reference spectra obtained at the same spectrometer using a mixture of 5 metabolites in each sample. The assigned peaks were integrated with MNova 10.0 (Metrelab Research).

### Data analysis

In general, data are presented as means ± SD. Insulin data are presented as means ± SEM. For all data types comparisons between different groups were carried out by ANOVA analysis, followed by Student’s paired *t* test using the GraphPad Prism version 6 (La Jolla, USA). Bonferroni-corrected *P-values* ≤ 0.05 were considered significant.

## Supplementary information

Supplementary information
