# Supplementary material for: Pancreatic β-cells respond to fuel pressure with an early metabolic switch
Source: Sci Rep. 2020 Sep 22;10:15413. doi: 10.1038/s41598-020-72348-1 (PMC7508987; doi:10.1038/s41598-020-72348-1)
Supplement: Supplementary file 1 — Supplementary information [file 41598_2020_72348_MOESM1_ESM.docx]

SUPPLEMENTARY INFORMATION

Pancreatic β-cells respond to fuel pressure with an early metabolic switch

**Ronja M. Malinowski^1^, Seyed M. Ghiasi^2^, Thomas Mandrup-Poulsen^2^, Sebastian Meier^3^, Mathilde H. Lerche^1^, Jan H. Ardenkjær-Larsen^1^, Pernille R. Jensen^1*^**

^1^Dept. of Health Technology, Technical University of Denmark, Kgs. Lyngby, Denmark
^2^Dept. of Biomedical Sciences, University of Copenhagen, Copenhagen, Denmark

^3^Dept. of Chemistry, Technical University of Denmark, Kgs. Lyngby, Denmark

To whom correspondence should be addressed:

Pernille R. Jensen,

Department of Health Technology,

Technical University of Denmark,

Oersteds Pl. Bldg. 349, Room 120, 2800 Kgs. Lyngby.

Telephone: (+45)45253688;

E-mail: peroje@dtu.dk.

Table of contents

1. Table S1. Additional information from literature survey (Fig. 1).

2. Figure S1. Glucose consumption.

3. Table S2. Cell viability.

4. Figure S2 Insulin content.

5. Table S3. Fitted metabolite amounts.

6. Figure S3. Metabolite ratios from HSQC spectra.

7. Figure S4. Relative metabolite amounts from HSQC spectra.

8. Figure S5. Schematic illustration of β-cell metabolism.

1. Table S1. Additional information from literature survey (Fig. 1).

**Table S1.** Additional information from references used in the literature survey represented in Fig 1. Three very similar buffers are used for the insulin secretion assay: **HBSS** (114 mM NaCl, 4.7 mM KCl, 1.2 mM KH_2_PO_4_, 1.16 mM MgSO_4_, 25.5 mM NaHCO_3_, 20 mM HEPES, 2.5mM CaCl_2_, and 0.2% BSA at pH 7.2). **KRBH** (114 mM NaCl, 4.4 mM KCl, 1.28 mM CaCl_2_, 1 mM MgSO_4_, 29.5 mM NaHCO_3_, 10 mM HEPES, and 0.1% BSA, pH 7.4), **KRB** (135 mM NaCl, 5 mM KCl, 1 mM MgSO_4_, 0.4 mM H_2_PO_4_, 20 mM HEPES).

| Refe-rence no. | Cell line | Incubation conditions | | | Measured parameters | | | |
| --- | --- | --- | --- | --- | --- | --- | --- | --- |
|  |  | Time  [h] | Glucose [mM] | Buffer | Insulin secretion | Metabolites | Glucose consumption | Other  parameters |
| 1 | INS-1 (832/13) | 48  48 | 2.8  16.7 | HBSS | x |  |  | Mitochondriamembrane potential, oxygen consumption rate. |
| 4 | INS-1 (832/13) | 1  1  1  1 | 2  4  12  20 | KRBH | x | x | x |  |
| 18 | INS-1 (832/13) | 4  4  4 | 3  6  12 | HBSS | x |  |  | Oxygen consumption rate, isotopomer analysis |
| 30 | INS-1E  BRIN-BD11 | 20 | 25 | KRBH | x | x |  |  |
| 31 | INS-1 (832/13) | 1 | 3  17 | HBSS | x | x |  | Oxygen consumption rate, HIF1-alfa |
| 32 | INS-1 (832/13) | 4 | 3  15 | n.d. | x |  | x | Tryphane blue viability, isotopomer analysis |
| 33 | INS-1 (832/13) | 48 | 3  17 | HBSS | x | x |  | Proteomics |
| 34 | INS-1E | 24  48 | 17 | KRB | x | x |  | p-ERK |
| 35 | INS-1 | 48  96 | 5  11  30 | KRBH | x |  |  | Gene expression of Small heterodimer partner |

2. Figure S1. Glucose consumption.


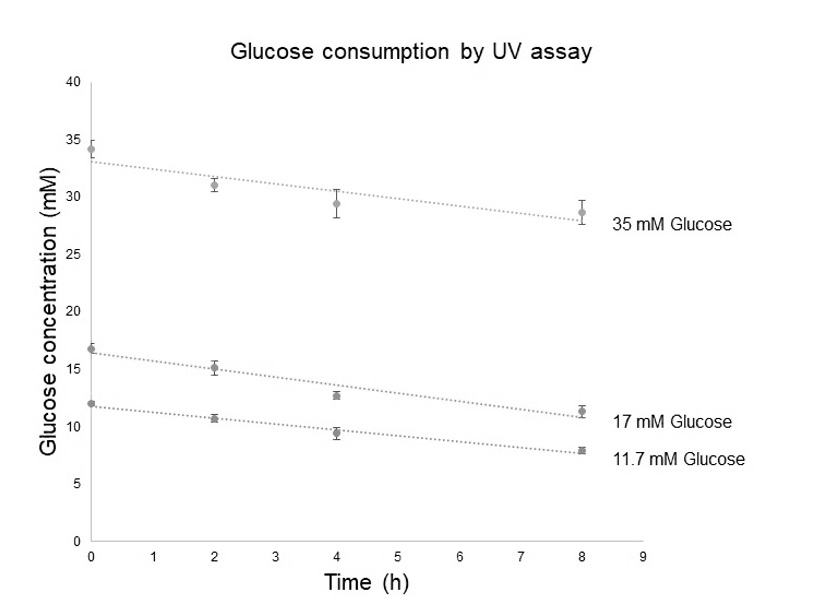


**Figure S1.** Glucose consumption during 8 hours incubation at start glucose concentrations of 11.7, 17 and 35 mM, respectively. The consumption was measured by a UV-assay based on peroxide production by glucose oxidase, n=4.

3. Table S2. Cell viability.

**Table S2.** Average cell viability during 8 hours of experiment under the three conditions of Figure S1, n=2. The viability was measured by an automatic cell counter (EVE, NanoEntek) as ratio between viable and total number of cells cells after trypan blue staining.

| Cell viability | | | |
| --- | --- | --- | --- |
| Glucose concentration | 2 hours | 4 hours | 8 hours |
| 11.7 mM | 94 % | 94 % | 90 % |
| 17 mM | 94 % | 94 % | 92 % |
| 35 mM | 93 % | 94 % | 94 % |

4. Figure S2 Insulin content.

**Figure S2.** Accumulated insulin content (n = 4) A) Corresponding to samples represented in Fig 3B (3, 11.7 and 17 mM for 4 hours). B) Corresponding to samples represented in Fig 5B (3, 11.7 and 17 mM for 2, 4 or 8 hours). No statistically significant change in insulin content was found for any condition (ANOVA analysis follow by Student’s t-test).

5. Table S3. Fitted metabolite amounts.

**Table. S3.** Average metabolite amounts measured by dDNP NMR. To these data, linear fits were applied as shown in main text Figures 6 A-C. The fitted slopes are used for the numbers presented under “Calculated”. *The slope in the obtained fits for pyruvate at 11.7 and 17 mM glucose are very similar (-0.26 nmol/min and -0.33 nmol/min, respectively), and here the average of these two determinations was used.

|  | **Measured average [nmol]** | | **Calculated* [nmol]** | |
| --- | --- | --- | --- | --- |
|  | Pyruvate | Lactate | Pyruvate | Lactate |
| 11.7 mM glucose |  |  |  |  |
| 2 h | 106 | 43 |  |  |
| 4 h | 51 | 107 | 70 | 80 |
| 8 h | 7 | 162 | 34 | 117 |
|  |  |  |  |  |
| 17 mM |  |  |  |  |
| 2 h | 183 | 46 |  |  |
| 4 h | 197 | 110 | 147 | 112 |
| 8 h | 76 | 244 | 111 | 178 |
|  |  |  |  |  |
| 35 mM |  |  |  |  |
| 2 h | 233 | 55 |  |  |
| 4 h | 287 | 174 | 456 | 172 |
| 8 h | 865 | 409 | 678 | 290 |

6. Figure S3. Metabolite ratios from ^1^H-^13^C HSQC spectra

A)

B)

**Figure S3.** Metabolite ratios from ^1^H-^13^C HSQC spectra are represented in Fig 8. The ratio represents the fold change for each metabolite between A) two time points (8 h/2 h) or B) two concentrations (35 mM/11.7 mM) with SEM for three biological replicates. The ratios vary around zero, which equals no change.

7. Figure S4. Relative metabolite amounts from ^1^H-^13^C HSQC spectra.

**Figure S4**. Estimate of relative amounts (± SEM) of metabolites from HSQC integrals (n=3). For this comparison each integral was divided by the number of protons in the atom group giving rise to the HSQC signal and all amounts were normalized to the most abundant metabolite Lactate. This estimate does not take response factors into account. Glycerol and fructose are discussed in the main text and are thus highlighted in white.

8. Figure S5. Schematic illustration of β-cell metabolism.

**Figure S5**. Schematic illustration of the β-cell metabolism that highlights the crossroads between glycolysis, the energy metabolism (TCA) and two fuel surfeit pathways; lipid synthesis and the polyol pathway. The enzymatic steps involving NADH and NAD^+^, respectively, are highlighted in red, together with the two enzymes using the reduced NADH; Glycerol-3phosphate dehydrogenase (G3PDH, 1) and lactate dehydrogenase (LDH, 2).
